# Reproductive outcomes of mosaic embryo transfers – rethinking the clinical relevance of contemporary mosaicism reporting in preimplantation genetic testing for aneuploidy

**DOI:** 10.1097/GCO.0000000000001101

**Published:** 2026-04-27

**Authors:** Christian Simon Ottolini, Silvia Caroselli, Antonio Capalbo

**Affiliations:** a Department of Maternal and Fetal Medicine, UCL Institute for Women’s Health, University College London, London, UK; b Department of Experimental Medicine, Sapienza University of Rome, Rome; c Department of Psychological Health and Territorial Sciences, School of Medicine and Health Sciences, ‘G. d’Annunzio’ University of Chieti-Pescara, Chieti; d Unit of Molecular Genetics, Center for Advanced Studies and Technology (CAST), ‘G. d’Annunzio’ University of Chieti-Pescara, Chieti, Italy

**Keywords:** constitutional aneuploidy, mosaic embryo transfer, preimplantation genetic testing for aneuploidy, reproductive potential

## Abstract

**Purpose of review:**

Next-generation sequencing–based preimplantation genetic testing for aneuploidy (PGT-A) has enabled the detection of intermediate copy-number signals, leading to widespread clinical classification of embryos as chromosomally ‘mosaic’. Mosaic embryo transfer (MET) is now routinely considered in assisted reproduction, yet the clinical relevance of mosaic classification remains uncertain. This review critically evaluates reproductive outcomes following MET in light of embryonic biology, technical constraints of PGT-A, and study design.

**Recent findings:**

Across the body of evidence, embryos labelled as mosaic retain substantial reproductive potential, with largely reassuring outcomes once implantation occurs. Apparent differences in reproductive efficiency, particularly among high-level mosaic categories, are better explained by study design bias, diagnostic imprecision, and classification artefact than by a clear biological intolerance to mosaicism. When selection bias and post hoc stratification are minimised, mosaic classification contributes little independent information beyond established clinical and embryological factors. The persistent signal observed in a small subset of embryos is increasingly attributable to misclassified constitutional aneuploidy rather than by adverse effects of mitotic mosaicism.

**Summary:**

These findings shift the conceptual framework away from mosaicism as a clinically actionable diagnosis and toward a model in which accurate identification of constitutional aneuploidy is the principal determinant of reproductive outcome.

**KEY POINTS**

- Embryos classified as mosaic by next-generation sequencing–based preimplantation genetic testing for aneuploidy (PGT-A) retain substantial reproductive potential and frequently result in healthy live births.
- Observational non-blinded studies reported reductions in mosaic embryo transfer efficiency that are more plausibly explained by study design, selection bias, and diagnostic imprecision.
- Prospective blinded non-selection studies show that intermediate copy-number findings add little independent clinical value once constitutional aneuploidy is excluded.
- High-level mosaic classifications are enriched for misclassified constitutional aneuploidy, providing a coherent explanation for adverse outcome signals in this subgroup.
- Future refinement of PGT-A should prioritise accurate discrimination of constitutional meiotic aneuploidy over reporting mosaicism inferred solely from intermediate copy-number variation.

## INTRODUCTION

The application of next-generation sequencing (NGS)–based preimplantation genetic testing for aneuploidy (PGT-A) has fundamentally reshaped embryo assessment in assisted reproduction. Compared with earlier cytogenetic platforms, the increased resolution of NGS has enabled the detection of intermediate copy-number signals from small trophectoderm biopsies, leading to the widespread clinical designation of embryos as chromosomally ‘mosaic’. Over the past decade, mosaic embryo transfer (MET) has evolved from an exceptional or experimental practice to a routine consideration in many IVF programs, particularly when euploid embryos are unavailable.

Debate surrounding MET has often focussed on whether embryos reported as mosaic are intrinsically less viable or pose increased risks to pregnancy or offspring. However, this framing obscures a more fundamental issue: mosaicism as reported by PGT-A represents a classification derived from quantitative copy-number thresholds applied to a limited biopsy, rather than a definitive diagnosis of embryo-wide chromosomal constitution. Consequently, differences in reproductive outcomes attributed to mosaicism may reflect limitations of measurement, study design, and classification rather than underlying embryonic biology. This review therefore examines the most influential published studies on the reproductive outcomes following MET in the context of (a) the biological origins of chromosomal mosaicism, (b) how mosaicism is detected and reported by PGT-A, and (c) the strengths and limitations of retrospective and prospective outcome studies.

## MITOTIC ERROR IN EARLY CLEAVAGE-STAGE DEVELOPMENT

Chromosomal mosaicism in human embryos arises from post-zygotic mitotic chromosome segregation error. Elegant work by Currie *et al*. (2022) [1] demonstrated that early cleavage-stage embryos are particularly prone to mitotic abnormalities, including multipolar divisions, lagging chromosomes, and chromatin bridges, events that can give rise to chromosomal mosaicism. These findings provided early biological evidence that chromosomal heterogeneity is a common feature of early human development rather than a rare pathological event.

Ottolini *et al*. (2017) [2] used high-resolution genomic analysis to show that abnormal cleavage patterns are strongly associated with gross mitotic segregation errors in blastomeres of developing embryos, including whole-chromosome and complex aneuploidies. Importantly, their work demonstrated that mitotic errors arising at early divisions can propagate into later developmental stages, even if karyotypically abnormal cells can arrest prior to blastocyst formation. Altogether, these studies establish a mechanistic link between early mitotic instability and the emergence and resolution of chromosomal mosaicism prior to the blastocyst stage.

## TROPHECTODERM BIOPSY AND INTERMEDIATE COPY NUMBER

In clinical practice, mosaicism has classically been inferred almost exclusively from quantitative copy-number data generated from a single trophectoderm biopsy. NGS-based PGT-A platforms infer chromosomal status by comparing read-depth across the genome following whole-genome amplification. Embryos are typically classified as euploid, aneuploid, or mosaic based on laboratory-defined copy-number thresholds, with intermediate values interpreted as evidence of mosaicism.

The clinical interpretation of intermediate copy-number signals as mosaicism is primarily supported by cell-mixing validation studies [3]. In these experiments, defined proportions of euploid and aneuploid cell lines were combined and analysed using NGS-based copy-number profiling, demonstrating that under controlled conditions, such platforms can distinguish mixtures containing varying proportions of abnormal cells. This analytical principle was subsequently extrapolated to clinical multicell trophectoderm biopsy samples. However, this translation occurred in the absence of robust validation using primary trophectoderm cells, clinically relevant biopsy conditions, or comprehensive embryo-level confirmation studies. As a result, the application of intermediate copy-number thresholds to infer embryonic mosaicism rests on analytical sensitivity rather than direct biological validation.

## AD HOC OBSERVATIONAL ANALYSIS OF MOSAIC EMBRYO TRANSFER DATA

The contemporary clinical understanding of outcomes following mosaic embryo transfer (MET) in non-blinded practice has evolved through a sequence of increasingly large and detailed studies. Victor *et al*. (2019) [4] provided the first systematic prospective clinical characterisation of MET outcomes. Mosaic embryos demonstrated meaningful reproductive potential, with implantation rates of ~40% and ongoing pregnancy/live birth rates of ~30–35%.

Zhang *et al*. (2020) [5] reported one of the earliest multicentre prospective cohort studies evaluating mosaic embryo transfers performed in cases where no euploid embryos were available and compared outcomes with an age-matched contemporary euploid embryo transfer cohort. Mosaic embryo transfer resulted in meaningful reproductive outcomes but with lower clinical pregnancy (40.6%) and ongoing/live birth rates (27.1%) and higher miscarriage rates (33.3%) than euploid embryo transfer (59.1, 47.0, and 20.5%, respectively). Importantly, all live births following mosaic transfer were chromosomally normal, with no confirmed cases of foetal mosaicism, consistent with prior reports.

Building on these early clinical experiences, Viotti *et al*. (2021) [6] reported the first large multicentre retrospective analysis of MET outcomes. At the cohort level, mosaic embryos exhibited lower implantation rates (46.5 vs. 57.2%), lower ongoing pregnancy/live birth rates (37.0 vs. 52.3%), and higher miscarriage rates (~20–25% vs. 8.6%) compared with euploid embryos. This study introduced detailed stratification by mosaic characteristics, demonstrating that for whole-chromosome mosaics, embryos with less than 50% intermediate copy number had significantly better outcomes than those greater than or equal to 50%, whereas for segmental mosaics, mosaic level had minimal impact. Increasing chromosomal complexity was associated with progressively poorer outcomes, from single-chromosome mosaics to complex profiles. Viotti *et al*. (2023) [7] further expanded this evidence base through analysis of the International Registry of Mosaic Embryo Transfers, with detailed obstetric and neonatal follow-up. Consistent with earlier findings, mosaic embryos showed reduced implantation and increased early pregnancy loss compared with euploid embryos. Crucially, among pregnancies progressing to delivery, neonatal outcomes were indistinguishable from those following euploid embryo transfer. In matched analyses, there were no differences in gestational age at delivery, birthweight, rates of preterm birth, or neonatal complications. Among 488 live-born infants, only one major congenital anomaly (0.2%) was reported, a rate below expected background population levels. Prenatal diagnostic data were available for 250 pregnancies following mosaic embryo transfer, of which 236 (94.4%) yielded normal results; only 3 pregnancies (1.2%) demonstrated persistence of the same chromosomal abnormality identified at the blastocyst stage.

Interestingly, a very recent publication continues to reinforce the reproducibility of findings from retrospective, non-blinded studies of mosaic embryo transfer as PGT-A technology continues to evolve. Jin *et al*. (2026) [8^■■^], using a propensity score–matched retrospective design in a large single-centre cohort, again demonstrated that mosaic embryo transfer results in meaningful pregnancy and live birth rates, with outcome heterogeneity largely confined to specific post hoc mosaic subcategories.

Collectively, these studies support the clinical transfer of embryos classified as mosaic, demonstrating meaningful live birth rates and largely reassuring obstetric and neonatal outcomes while suggesting variation in reproductive efficiency that favours de-prioritisation rather than exclusion within an embryo cohort. However, the supporting evidence is derived entirely from observational, non-blinded studies and does not establish a causal link between mosaic classification and reduced reproductive potential relative to euploid embryos. Mosaic embryos are typically transferred only in the absence of euploid embryos, introducing substantial confounding by indication. Interpretation is further limited by post hoc stratification and reliance on mosaic classification based on intermediate copy-number thresholds from a single trophectoderm biopsy. Within this context, reported reductions in implantation or increases in early pregnancy loss are more plausibly explained by selection bias and diagnostic uncertainty than by an intrinsic biological intolerance of mitotic mosaicism.

## PROSPECTIVE BLINDED STUDIES ASSESSING MOSAIC EMBRYO TRANSFER OUTCOMES

In contrast to retrospective or observational studies, the first indication putative mosaicism findings might lack independent predictive value came from a small prospective, blinded, non-selection study for aneuploidy in PGT-A reported by Wang *et al*. (2021) [9]. Among the 25 embryos transferred that were subsequently classified as mosaic, 11 resulted in live birth (44.0%), compared with 56 live births among 110 embryos classified as euploid (50.9%) (Fisher’s exact test, *P* = 0.66).

Capalbo *et al*. (2021) [10] conducted a larger prospective, double-blinded, non-selection trial specifically designed to assess outcomes after single-embryo transfer of embryos classified as euploid, low-level mosaic (20–30% intermediate copy number), or medium-level mosaic (30–50%). Live birth rates were equivalent across groups, at 43.4% for euploid, 42.9% for low-level mosaic, and 42.0% for medium-level mosaic embryos, meeting predefined non-inferiority criteria. Miscarriage rates were also similar (12.0, 11.0, and 12.7%, respectively), with no increase in pregnancy loss after confirmation of foetal cardiac activity. Obstetric outcomes, gestational age at delivery, and birthweight did not differ between groups. Prenatal and postnatal genetic testing, where performed, revealed no cases of foetal mosaicism, uniparental disomy, or chromosomal abnormality attributable to mosaic embryo transfer.

Using the same, prospective, blinded, non-selection design, the recent work from Gill *et al*. (2025) [11^■^] was not restricted to low- or moderate-level mosaic embryos, instead evaluating outcomes across the full spectrum of embryos reported as mosaic, including high-level mosaic embryos. Mosaic embryos demonstrated a live birth rate of 53.2% (749/1407) compared with 60.0% (5052/8421) for embryos reported as euploid [adjusted odds ratio (OR): 0.79, 95% confidence interval: 0.70–0.89]. Stratification by mosaic level showed that this difference was not uniform across mosaic categories and was largely attributable to embryos classified as high-level mosaic (≥50%), which represented a small minority of transfers (372 embryos; 3.8%) and had a live birth rate of 46.5% (adjusted OR 0.61, *P* < 0.01). In contrast, embryos classified as low- or moderate-level mosaic (<50%), comprising the majority of mosaic transfers (1035 embryos; 10.5%), exhibited live birth rates comparable to euploid embryos after multivariable adjustment.

Earlier reproductive endpoints followed a similar pattern. Implantation rates were 65.1% for euploid embryos, 63.4% for low-/moderate-level mosaic embryos, and 55.2% for high-level mosaic embryos, with statistically significant differences observed only for the high-level mosaic group (*P* < 0.01). Miscarriage rates were not significantly different across groups (12.4% euploid, 12.7% low-/moderate-level mosaic, 13.1% high-level mosaic; all *P* > 0.05), and importantly, no excess pregnancy loss was observed after confirmation of foetal cardiac activity. Analysis by mosaic type further demonstrated that segmental mosaic embryos showed outcomes closely aligned with euploid embryos, whereas whole-chromosome mosaic embryos accounted for most of the observed reduction in reproductive efficiency.

Perinatal and neonatal outcomes were reassuring and broadly equivalent across groups. Among 6650 live births, there were no statistically significant differences between mosaic and euploid embryo transfers in gestational age at delivery, mean birthweight, rates of preterm birth, or NICU admission. Rates of congenital anomalies were low and comparable (1.5% following mosaic embryo transfer vs. 1.3% following euploid transfer; *P* > 0.05), with no evidence of enrichment for chromosomal abnormalities among infants born following mosaic embryo transfer.

Crucially, Gill *et al*. extended beyond outcome reporting to formally assess the clinical utility of mosaic embryo reporting for embryo selection. Predictive modelling incorporating standard clinical and embryological variables demonstrated that inclusion of mosaic embryo status did not meaningfully improve discrimination for live birth, with the area under the receiver operating characteristic curve increasing only from 0.552 to 0.555, a difference that was not statistically significant. Decision-tree and multivariable analyses similarly failed to identify mosaic status as a dominant predictor of outcome. Taken together, these data demonstrate that, even in a dataset sufficiently powered to detect small statistical differences, routine reporting of mosaicism does not improve the identification of embryos with the highest reproductive potential, and that established clinical and embryological factors exert substantially greater influence on outcomes than mosaic classification alone.

## THE MISCLASSIFICATION OF HIGH-LEVEL MOSAICISM AND TOWARD IMPROVED DISCRIMINATION OF MEIOTIC ANEUPLOIDY IN PREIMPLANTATION GENETIC TESTING FOR ANEUPLOIDY

Where both retrospective observational studies and prospective, blinded, non-selection studies converge is the persistent, albeit modest, signal that embryos classified as ‘high-level mosaic’ tend to show inferior reproductive outcomes compared with euploid embryos and lower-level mosaic categories. Importantly, this convergence does not imply a causal biological intolerance of mitotic mosaicism but instead aligns closely with accumulating biological and technical evidence that high-level mosaic calls are enriched for misclassified constitutional chromosomal abnormalities.

Multifocal biopsy studies provide a critical biological context for interpreting this observation. In Capalbo *et al*. (2021) [10], embryos initially reported as mosaic on a single trophectoderm biopsy underwent extensive re-analysis using multiple biopsies from different trophectoderm regions and the inner cell mass. While embryos classified as low- or moderate-level mosaic were overwhelmingly euploid across the remainder of the embryo, a substantial proportion of embryos classified as high-level mosaic were uniformly aneuploid in all sampled regions, with no evidence of true cellular mosaicism.

Popa *et al*. (2025) [12^■^] directly addressed the origin of mosaic embryo calls by re-analysing embryos labelled as mosaic using single-nucleotide polymorphism (SNP) genotyping and karyomapping to distinguish meiotic from mitotic error. Among all embryos reported as mosaic, 32.6% (46/141) harboured a meiotic-origin abnormality, with this proportion rising sharply to 55% (43/78) among high-level mosaic embryos, compared with only 4.7% (3/63) among low-level mosaic embryos. This quantitative gradient provides a coherent mechanistic explanation for why high-level mosaic categories, although rare at the population level, may consistently show reduced implantation or live birth rates in observational cohorts and even a modest signal in large blinded studies. As the mosaic level increases, the category becomes increasingly ‘contaminated’ by embryos with constitutional aneuploidy, which is known to intrinsically compromise reproductive potential.

With the aim to reliably distinguish meiotic (constitutional) aneuploidy from non-meiotic sources of intermediate copy-number signal, several groups have begun to incorporate SNP-based analysis and parental or reference genomes into PGT workflows to directly infer the origin of chromosomal abnormalities [13–17]. By leveraging haplotype information, these approaches can determine whether an aneuploidy arose during meiosis or post-zygotically and, in many cases, identify the parental homolog involved. Importantly, this strategy aims not simply to refine copy-number resolution, but to reframe embryo classification around biologically meaningful distinctions, separating embryos with constitutional meiotic errors, known to compromise reproductive potential, from those showing intermediate copy-number patterns not of meiotic origin. This would allow future trials to finally determine whether mitotic aneuploidy detected at the blastocyst stage carries independent reproductive consequences, or whether it represents a largely tolerated feature of early human development. This shift reflects a broader conceptual evolution in the field, moving away from increasingly granular interpretation of intermediate copy-number values and toward more robust identification of clinically relevant abnormalities.

## CONCLUSION

Collectively, evidence from the most influential published studies on mosaic embryo transfer demonstrates that embryos classified as mosaic by NGS-based PGT-A retain substantial reproductive potential and frequently result in healthy live births. Apparent reductions in reproductive efficiency reported in some studies are more plausibly attributable to study design, selection bias, and diagnostic imprecision than to an inherent biological intolerance of mitotic mosaicism. Prospective blinded non-selection studies indicate that intermediate copy-number findings provide little independent clinical value for embryo selection once constitutional aneuploidy has been excluded. Convergent biological evidence further suggests that high-level mosaic classifications are enriched for misclassified constitutional aneuploidy, providing a coherent explanation for the outcome signals consistently observed in this subgroup. Future refinement of PGT-A should build on its established clinical utility by improving the distinction between constitutional aneuploidy of meiotic origin and non-meiotic sources of intermediate copy-number variation. Continued technological advances that better resolve the origin of chromosomal abnormalities have the potential to enhance understanding of the reproductive potential of embryos with true embryonic mosaicism, support ongoing prospective evaluation, and refine clinical interpretation while preserving the demonstrated benefits of PGT-A in embryo selection. Until such refinements are broadly implemented, the prevailing evidence supports cautious interpretation and, where accurate discrimination of constitutional aneuploidy has been validated, non-reporting of mosaicism inferred solely from intermediate copy-number results.

## Acknowledgements

None.

## Financial support and sponsorship

None.

### Conflicts of interest

There are no conflicts of interest.
